# Natural and adaptive IgM antibodies in the recognition of tumor-associated antigens of breast cancer (Review)

**DOI:** 10.3892/or.2015.4095

**Published:** 2015-06-30

**Authors:** MARIANA DÍAZ-ZARAGOZA, RICARDO HERNÁNDEZ-ÁVILA, RUBÍ VIEDMA-RODRÍGUEZ, DIEGO ARENAS-ARANDA, PEDRO OSTOA-SALOMA

**Affiliations:** 1 Departamento de Immunología, Instituto de Investigaciones Biomédicas, Universidad Nacional Autónoma de México, Ciudad Universitaria, 04510 México; 2 Unidad de Investigación Médica en Genética Humana, Centro Médico Nacional, Siglo XXI, Instituto Mexicano del Seguro Social, 06729 México; 3 Posgrado en Ciencias Biológicas, Universidad Nacional Autónoma de México, Ciudad Universitaria, 04510 México, DF, México

**Keywords:** breast cancer, natural IgM, adaptive IgM, TAA, TACA, early diagnosis

## Abstract

For early detection of cancer, education and screening are important, but the most critical factor is the development of early diagnostic tools. Methods that recognize the warning signs of cancer and take prompt action lead to an early diagnosis; simple tests can identify individuals in a healthy population who have the disease but have not developed symptoms. Early detection of cancer is significant and is one of the most promising approaches by which to reduce the growing cancer burden and guide curative treatment. The early diagnosis of patients with breast cancer is challenging, since it is the most common cancer in women worldwide. Despite the advent of mammography in screening for breast cancer, low-resource, low-cost alternative tools must be implemented to complement mammography findings. IgM is part of the first line of defense of an organism and is responsible for recognizing and eliminating infectious particles and removing transformed cells. Most studies on breast cancer have focused on the development of IgG-like molecules as biomarkers or as a treatment for the advanced stages of cancer, but autoantibodies (IgM) and tumor-associated antigens (proteins or carbohydrates with aberrant structures) have not been examined as early diagnostic tools for breast cancer. The present review summarizes the function of natural and adaptive IgM in eliminating cancer cells in the early stages of pathology and their value as early diagnostic tools. IgM, as a component of the immune system, is being used to identify tumor-associated antigens and tumor-associated carbohydrate antigens.

## 1. Introduction

The detection of tumors at early stages allows curative treatment to be administered before tumor progression occurs (1); consequently, patients live longer and fare better than those with advanced cancer (2,3). The detection of such tumors is challenging, which is particularly significant in the high-risk population, in whom the incidence of disease is higher (1,4). Major components of the early detection of cancer are education and screening, but the most important aspect is the development of early diagnostic tools. Tools that recognize the warning signs of cancer so prompt action can be taken may ensure an early diagnosis, and simple tests can identify individuals in a healthy population who have the disease but have not developed symptoms (5). Diagnoses that are based on symptoms are unacceptable for cancer, as they usually appear when the tumors are sufficiently large in size (6).

An early diagnosis is paramount in breast cancer (BC), since it is the most frequent tumor occurring in women in industrialized and developing nations (2,6). Thus, early detection remains the cornerstone of controlling BC to improve patient outcomes and survival. Breast tumors express aberrant levels of mutated or modified forms of proteins that are associated with malignant growth. These proteins, called tumor-associated antigens (TAAs) and tumor-associated carbohydrate antigens (TACAs), are able to stimulate cellular and humoral immune responses; TAAs are identified by serum antibodies (Abs) of patients (7,8).

The only effective screening method for BC is mammography. Mammography is expensive and is only cost-effective and feasible in developed countries with good health infrastructure. Many low- and middle-income nations must implement low-cost screening, such as clinical breast examination and early diagnostic tools (5,9). Ongoing studies are evaluating inexpensive screening methods that can be implemented and sustained in low-resource settings, based on the detection of antitumor antigens by immunoglobulin M (IgM) Abs in the serum of female mice with BC. In these studies, the patterns of antigen (Ag) recognition by Abs in 2D immunoblots are identified and expressed as immunological signatures, allowing certain patterns to be correlated with resistance or susceptibility (10).

Two types of IgM exist: natural, which is present in an organism without prior antigenic contact and is part of the first-line defense; and adaptive, which develops after antigenic challenge (11). Natural IgM also has a significant function in maintaining tissue homeostasis, promoting the phagocytic clearance of apoptotic cells and preventing infectious and autoimmune diseases (12), and in recognizing and removing precancerous and cancerous cells (13–18).

In the present review, we discuss the function of natural IgM and adaptive IgM in eliminating cancer cells in the early stages of BC and their potential as early diagnostic tools and how, as components of an organism's defense, they can be used to identify TAAs and TACAs.

## 2. IgM antibodies

IgM, which has *μ* heavy chains, is the first class of antibody that is synthesized by and appears on the surface of a developing B cell, although many B cells eventually switch to other classes (19). It is also the major class that is secreted into the blood in the early stages of a primary antibody response on initial exposure to an Ag.

IgM is the first line of defense of an organism. In its secreted form, IgM is a pentamer that comprises 5 4-chain units, giving it a total of 10 Ag-binding sites and thus higher valency than the structures of other immunoglobulins (Igs) and allowing it to bind Ags with high avidity (20). Each pentamer contains one copy of another polypeptide chain, called a J (joining) chain (21). IgM regulates B cell development (22), facilitates the clearance of apoptotic cells (23), modulates inflammatory responses (24) and autoimmune diseases (25) and mediates the elimination of cancer cells (13).

The binding of an Ag to a single secreted pentameric IgM molecule initiates the complement system. When the Ag resides on the surface of an invading pathogen, senescent cells, cell debris, or precancerous or cancer cells, this activation marks pathogens and transformed cells for phagocytosis or kills them directly (21).

### Natural IgM antibodies

Natural Abs are predominantly IgM and to a lesser extent IgA and IgG (26–28) and are polyreactive and of low affinity (29). Natural IgM circulates in healthy individuals in the absence of exogenous antigenic stimulation or Ag-driven selection (30,31). Natural IgM levels in the serum of newborns and in animals that are grown under sterile conditions on an Ag-free diet do not differ from those of normal animals (11). Natural IgMs are also in humans (32).

Natural IgM has a significant function in primary defense mechanisms (14,33,34). They participate in the early recognition and elimination of bacterial and viral invaders and altered self-material from an organism, reacting with cell surface receptors and recognizing and removing apoptotic and senescent cells, cell debris and self-Ags (13,33,35–37). Natural IgM auto-Abs help suppress pathogenic IgG auto-Ab responses (38).

Natural IgM is associated with the recognition and removal of precancerous and cancerous cells (13–18). Natural IgM binds preferentially to post-transcriptionally modified cell surface Ags that are tumor-specific, recognizing the conserved structures of carbohydrate epitopes (14,39–42). Carbohydrate epitopes that are recognized by natural IgM are stably expressed in many tumors at various precursor stages. Unlike epitope-based single-peptide chains, glycoepitopes share structural homologies beyond the limits of the protein families; thus, they can crossreact and constitute the preferred targets for natural IgM Abs (35).

Natural IgM is produced by a small subset of B1 cells (CD5^+^) and B cells in the marginal zone (Mz) and do not require affinity maturation to provide early protection (43). B1 cells are B220^low^IgM^hi^CD23^low/−^CD43^+^IgD^low^, have the characteristics of activated cells, and have greater size and cytoplasmic complexity than B2 cells (44).

Natural IgM Abs are germline-encoded and not affinitymatured. Over 80% of natural IgM Abs are expressed by VH genes of the VH3 family (45) and have low affinity (kDa = 10^−4^ to 10^−7^ mol^−1^) (46). The strength of the Ag-Ab interaction is enhanced by the potency of IgM in engaging the complement pathway; unlike IgG, a single IgM molecule can bind to C1q and activate the complement cascade (18). Natural IgM is equipped with a λ chain, unlike other Abs (14).

### Adaptive IgM antibodies

Adaptive IgM is the first antibody to appear after an immunological challenge, but its production normally falls during the development of the IgG response. Consequently, IgM is generally not considered to have a significant function in long-term immunity, although it is effective in host defense (19,47).

Long-lasting humoral immunity is typically associated with the development of high-affinity Ab and isotype switching (47). For example, IgM that is induced by immunization differs from natural IgM with regard to its structure in the Ag-binding centers, affinity, specificity repertoire and spectrum of functions (11,48). These IgM Abs constitute a small fraction of circulating molecules, are monoreactive, and have higher affinity (10^−7^ to 10^−11^ mol^−1^), and their variable regions contain point mutations. The half-life of monoreactive IgM is 35 h (45,46).

Adaptive IgM is produced by B2 cells and follicular B cells, which are typical of the adaptive immune response. B2 cells mediate T-dependent reactions of the germinal center (GC) and effect the development of memory cells and highaffinity plasma cells (29). Mature B2 cells produce Ab after being stimulated, expanded, and selected in GCs in the presence of T helper (Th) cells; thus, they are important in the adaptive immune response, representing the first-line defense against most infections and the only form of protection against encapsulated bacteria. The adaptive immune response requires at least 1 week to produce monospecific high-affinity Abs, first generating IgM and then isotype-switching to IgG (49). B2 cells have a B220^hi^IgM^int^CD23^hi^IgD^hi^ phenotype (44).

**Figure 1:**
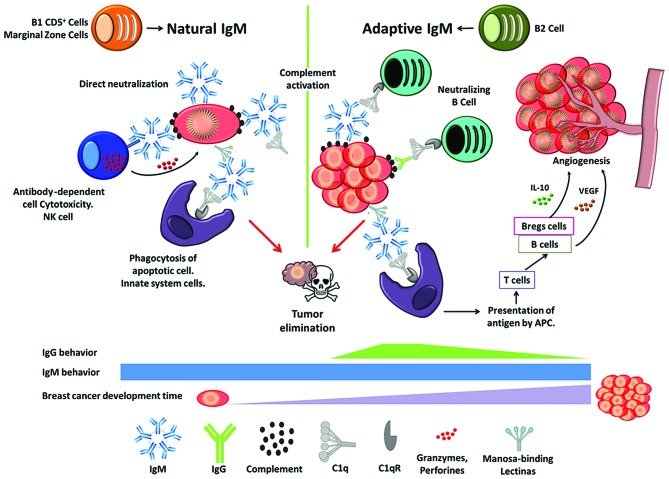
Natural IgM is produced by B1 cells and marginal zone cells, and adaptive IgM is synthesized by B2 cells. Both types of IgM have several functions in the immune response, eliminating tumor cells when they begin to transform (natural IgM) and grow (adaptive IgM). But, when a tumor is established, components of the immune system, such as B cells and adaptive regulatory B cells (Bregs), secrete vascular endothelial growth factor (VEGF) and IL-10, respectively, promoting angiogenesis, inhibiting T cell responses, and accelerating progression, all of which facilitate the spread of neoplastic cells. IgG is present in the early stages of breast cancer but becomes immunosuppressed over time, whereas IgM remains constant.

Fig. 1 shows the various functions of natural and adaptive IgM, as identified in murine models, and how IgM and IgG behave throughout the development of a breast tumor. Natural and adaptive IgM levels are constant from transformation until the tumor is established. Whereas IgG is present only in the initial stages of adaptive immunity, it becomes immunosuppressed when the breast tumor is formed. In innate and adaptive immunity, natural IgM and adaptive IgM, respectively, protect the organism from pathogenic infection, cellular debris, senescence, and transformed cells using many strategies, such as classical complement activation with C1q (50). IgM has been proposed to bind tightly to complement factor C1q and activate the complement cascade (51). It also neutralizes (52–54) and clears apoptotic cells by phagocytosis (55), binding to mannose-binding lectin (MBL), which interacts with apoptotic cells (56), and directs the clearance of immune complexes by binding to the putative Fca/mR receptor on phagocytes.

Natural and adaptive IgM molecules participate in the recruitment of Ags into secondary lymphoid organs, priming subsequent adaptive immune response (48,57). This mechanism forms the link between the innate and adaptive immune systems. In adaptive immunity, after IgM appears, T and B cells are activated, and adaptive regulatory B cells (Bregs) develop, the immunological mechanisms are damaged, and the relationship between the tumor and immune response shifts toward a state of conditioned immunosuppression (58,59), causing late immune responses to fail to develop strategies that eliminate tumor cells. B cells in the inflammatory infiltrate effect the release of vascular endothelial growth factor (VEGF), promoting angiogenesis and thus accelerating the spread of neoplastic cells through the lymphatics to regional lymph nodes (60). In addition, Bregs produce IL-10, which has suppressive effects on systemic immunity, inhibits T cell responses, and favors the induction of proinflammatory factors and angiogenic molecules (58,61).

## 3. Immune surveillance: Mechanism to eliminate cancer cells

In 1909, Paul Ehrlich postulated that the immune system not only eliminates pathogenic bacteria but also suppresses the growth of carcinomas with great frequency by generating Abs against malignant cells (35). Fifty years later, Burnet and Thomas revised the topic of natural immune protection against cancer. Burnet proposed that immune tolerance, with regard to tumor cell-specific neo-Ags, could effect an immunological reaction that prevents the development of cancers, defined as the immune surveillance concept (62,63). Transformed cells are removed by immune surveillance, comprising an immediate immune response that provides Abs against malignant cells and a secondary inherited immune response in which B cells are derived (35,43,64,65). The Ab response is known as the humoral arm of the immune system and is a critical mechanism in the primary and secondary responses against all types of nonself (bacteria, viruses, fungi and cancer) (15).

Innate immunity is the first line of defense and stimulates secondary adaptive immune responses (66), relying on Toll-like receptors (TLRs), which recognize pathogen-associated molecular patterns. These specific patterns are conserved and repetitive structures, such as carbohydrates on glycoproteins and glycolipids (eg, lipopolysaccharides) that are expressed independently of mutational events (67) and detected independently of T cells (35).

## 4. Autoantibodies and tumor-associated antigens in breast cancer

BC is a heterogeneous disease with tumors that express a variety of aberrant proteins (68). Natural and adaptive IgM can perceive foreign TAAs that undergo post-translational modifications, and natural IgM mediates the destruction of tumor tissues that recognize TACAs (69). The presence of post-translational modifications, such as glycosylation, phosphorylation, oxidation, and proteolysis, can induce immune responses by generating a new epitope, inducing its presentation by major histocompatibility complex (MHC) molecules and stimulating T cell receptors (70,71). Such modified proteins are wrongly localized, mutated, insufficiently folded, or aberrantly expressed and are associated with carcinogenic processes (eg, cell cycle progression, signal transduction, proliferation and apoptosis) (70,71). Cell surface glycans that are secreted into the serum by malignant cells provide a mechanism of tracking tumor burden. Many malignant cells, but not normal cells, overexpress CD20, ECFR and HER2, rendering them commonly used diagnostic markers, but not for early diagnosis (42,72).

Aberrant proteins have been used since the 1970's in serological studies in BC patients that have suggested that some portion of positive sera contains Abs that are directed toward TAAs, but they did not specify the type of Ab. The serum of 28 patients with BC showed positive fluorescence against breast carcinoma cells that were grown by tissue culture, while that of donors was negative (73). In another study, the serum from BC patients was tested for reactivity to a human breast tumor cell line; 45% of patients had complement-fixing Abs compared with 13% that had benign breast disease (74). In contrast, the serum of 55 BC patients of all ages and stages of disease harbored significantly elevated IgA levels and decreased IgG content vs. The control group (75). Ig levels are significantly lower in breast tissue compared with benign tissue, except for adaptive IgM, which is consistently higher in BC tissue of patients with stage I and II disease (76).

There are several blood tests that identify tumor Ags at high levels in patients with metastatic disease, but they are too insensitive for use in the early detection and diagnosis of BC (68). TAAs have modulate transmembrane signaling, which is required for proliferation, invasion and metastasis of tumor cells (72). Natural and adaptive IgM against TAAs can be measured as an early sign of BC *in vivo* and detect the disease earlier than current methods; furthermore, natural IgM is detected in the asymptomatic stages of cancer, up to 5 years before disease onset (77). Although there are IgG-based diagnostic biomarkers for BC that are under development and although hundreds of self-Ags and TAAs that are recognized by auto-Abs have been identified, no definitive Ab-based sero-logical markers for the early diagnosis of BC exist.

Auto-Abs against TAAs in the serum of BC patients can be easily detected and are inherently stable, persisting in the serum for long periods, since they generally do not undergo proteolysis, as do other polypeptides (71).

## 5. IgM antibodies directed against breast cancer tumor antigens

Both IgM types must be considered to develop a tool for an early diagnosis once the tumor has been established, since natural and adaptive IgM has direct cytotoxic effects on tumor cells. Certain IgM Abs have been isolated from the tumors of patients, since they eliminate tumors by inducing apoptosis *in vivo* (25,43) through the domain-independent pathway of cell death, binding to surface receptors that induce cell stress (13). Following, we discuss several types of IgM Abs that are used in the diagnosis of BC.

### FC-2.15

FC-2.15 is a murine monoclonal IgM Ab that was raised against human BC. FC-2.15 recognizes BC cells and certain normal cells, such as peripheral polymorphonuclear granulocytes (PMNs); specifically, the carbohydrate moiety of certain glycoproteins mediates the *in vitro* lysis of Ag-2.15^+^ cells by human complement. FC-2.15 induces antitumor responses and reversible neutropenia. In an analysis of epitope specificity, FC-2.15 specifically recognized terminally exposed Lewis^x^ trisaccharide but not sialyl-Lewis^x^, Lewis^a^, trifucosylated Lewis^y^, blood groups Ag A and B, globo H, or gangliosides. Lewis^x^ in its mostly O-linked is present in BC cells.

In contrast to other monoclonal Abs against carbohydrates, which have affinity constants of 10^3^ to 10^5^ M^−1^, FC-2.15 has an affinity constant of 6.9×10^7^ M^−1^, which might explain its potent effects *in vivo*. The presence of Lex epitopes on BC cells and peripheral PMNs explains the antitumor responses and neutropenia that were observed in a trial of FC-2.15. Neutropenia was inconsequential to the patients, although >90% of PMNs disappeared from the peripheral blood, and the neutropenia resolved rapidly after mAb infusion was halted, with the appearance of juvenile myeloid forms after repeated courses of mAb, as myeloid precursors are not lysed by FC-2.15 (78).

### Natural IgM antibody SC-1

The natural IgM Ab SC-1 was isolated from a patient with signet-ring cell carcinoma of the stomach (79). SC-1 binds to a tumor-specific carbohydrate epitope of decay acceleration factor-B (DAF; also called CD55), which is specifically expressed in the membrane of stomach carcinoma cells, and induces apoptosis by crosslinking the receptor *in vitro* and in experimental *in vivo* systems (35,80). The apoptotic effects of two novel sorafenib analogs, SC-1 and SC-43, in eliminating BC cells were examined. Sorafenib, SC-1 and SC-43 induced apoptosis, concurrent with downregulation of p-STAT3 and its downstream proteins, cyclin D1 and survivin, dose-dependently in BC cell lines (HCC-1937, MDA-MB-468, MDA-MB-231, MDA-MB-453, SK-BR3 and MCF-7). SC-1 and SC-43 also stimulated apoptosis through SHP-1-dependent STAT3 inactivation and had more potent apoptotic effects than sorafenib in human BC cells (81).

### Monoclonal IgM antibody PAM-1

The fully human germline-encoded monoclonal IgM Ab PAM-1 was isolated from a patient with gastric carcinoma. The blockade of growth factor receptors, such as EGFR and FGFR, which are often overexpressed in malignant cells, led to starvation and cell death. PAM-1 binds to CFr-1 (cysteine-rich fibroblast growth factor receptor). The post-transcriptionally modified CFr-1/PAM-1 receptor is expressed in nearly all epithelial cancers of every type and origin and in the precursor stages but not in healthy tissue. The binding of PAM-1 induces apoptotic events *in vitro* and *in vivo* (35,80).

CFR-1/PAM-1 receptor expression in the precancerous stages of BC was analyzed by immunohistochemistry and compared with normal breast tissue and adenocarcinomas. The CFR-1/PAM-1 receptor was expressed in nearly all precancerous stages and carcinomas, whereas normal breast tissue was negative. The unique expression of this CFR-1/PAM-1 receptor renders PAM-1 Ab an ideal diagnostic tool and therapeutic agent for precancerous and cancerous epithelial lesions in BC (16).

### Natural IgM antibody SAM-6

The levels of cell surface-associated chaperone GRP78 are high in BC cells. GRP78 is a ubiquitously expressed member of the heat-shock protein 70 (HSP70) family and governs cellular homeostasis by preventing stress-induced apoptosis. In malignant cells, which are permanently exposed to environmental stress, GRP78 is overexpressed, and its levels increase in the cytoplasm and on the cell membrane (41). Thus, GRP78 promotes tumor proliferation, survival, metastases and resistance to many therapies.

The fully human monoclonal IgM Ab SAM-6 binds to a new variant of GRP78, which has a molecular weight of 82 kDa. The epitope is an O-linked carbohydrate moiety that is specific to malignant cells (13). SAM-6 is internalized through endocytosis and mediates the lethal accumulation of oxidized lipoproteins, followed by apoptosis. Modified protective molecules, such as GRP78-SAM-6, are excellent targets for specific Abs that can neutralize the protective effects of tumor cells, disable mechanisms of drug resistance, and directly kill cancer cells by inducing apoptosis (13,41).

SAM-6 induces apoptosis and the accumulation of neutral and polar lipids in tumor cells but not normal cells. The non-physiological intracellular accumulation of neutral lipids, such as triglycerides and cholesterol, is cytotoxic and can lead to lipoptosis (35,65). SAM-6 binds to a cell surface receptor on malignant cells and oxidized low-density lipoprotein (LDL). Shortly after the internalization of Ab/oxidized LDL/receptor complexes and the formation of lipid depots, cytochrome *c* is released by mitochondria and subsequently, the initiators caspase-8 and caspase-9 and effectors caspase-3 and caspase-6 are activated. Thus, SAM-6 induces a near-intrinsic form of apoptosis by overfeeding malignant cells with lipoproteins (80,82).

### Murine monoclonal IgM antibody 3EL.2

Mammary serum Ag (MSA) belongs to the molecular family of breast mucins (MUCs) and is a macromolecular glycolic protein with a molecular weight of >300,000 kDa. MSA is targeted by the murine monoclonal IgM Ab 3EL.2. The diagnostic value of MSA in identifying BC has been studied in 56 healthy patients and 43 subjects with benign BC, in whom this Ag was abnormally elevated. Thus, 3EL.2 is useful in the clinic, is a good indicator of the extent of disease, and might have significant prognostic value (83).

### P10s

Abs that recognize such glycosphingolipids (GSLs) as GD2, GM2 and Lewis^Y^ (LeY) mediate complement-dependent cytotoxicity and have been suggested to be more cytotoxic to tumor cells than Abs that recognize proteins Ag or TACAs (84), which kill tumor cells by Ab-dependent cellular cytotoxicity. Carbohydrate mimetic peptides (CMPs) of TACAs induce IgM that targets TACAs in BC.

Preexisting ganglioside-reactive IgM has been detected in normal healthy individuals. Circulating gangliosides from tumors might be perceived as danger signals by the host's immune system, as evidenced by the endogenous antiganglioside immune response to gangliosides. Then, endogenous IgM against gangliosides might facilitate the elimination of these signals in BC in order to restore the immune competence of the host (69).

P10s-WRYTAPVHLGDG, a CMP that induces primarily weak anti-GD2 IgM responses that are crossreactive with several gangliosides, including GD3, GM2 and GD1a, has been developed. P10s was derived from a sequence (P10-GVVWRY-TAPVHLGDG) that was selected by panning a peptide library against the GD2-binding mAb ME36.1. The P10 peptide mediates antitumor responses (85). This sequence was further optimized by molecular modeling to overlap its binding interface more with the ME36.1 paratope, thus yielding P10s (86).

### Anti-mucin IgM

MUCs are highly glycosylated proteins that are expressed in cancers of epithelial origin in an underglycosylated form and have been used to develop several tests for cancer detection. MUCs are components of mammary cell-cell junctions and mediate ICAM-1-initiated signal transduction. Polymorphic epithelial mucin (PEM, or MUC1 with different epitopes: CA 15.3 and CA 27.29) and MUC16 (CA 125) are the most extensively studied MUCs, although the latter is more frequently used for ovarian cancer than for BC.

Recent evidence indicates that MUC-1 Ag induces apoptosis in T-lymphocytes, providing insight into the mechanisms of escape from immune surveillance by tumors (87).

MUC1 was examined to determine the incidence of naturally occurring MUC1 Ab in patients with early BC and correlate these Abs in pretreatment serum to disease outcome. IgG and IgM against MUC1 were measured by ELISA in pretreatment serum samples from 154 patients with BC and 302 controls. A positive test result for both anti-MUC1 IgG and IgM in pretreatment serum was associated with a significant benefit for disease-specific survival in BC patients. Patients with early BC with a natural humoral response to MUC1 are less likely to develop metastases and have better disease-specific survival. MUC1 Abs may control hematogenic tumor dissemination and outgrowth by aiding in the destruction of circulating or established MUC1-expressing tumor cells (88,89).

MUC1 is a high-molecular-weight molecule with multiple tandem repeats (VTSAPDTRPAPGSTAP-PAHG). The most immunogenic motif is APDTRPA, which harbors the epitope that is recognized by various monoclonal Abs, normal sera and cytotoxic T-cells. Mice that are vaccinated with MUC1 peptide that contains 1.5 tandem repeats and is conjugated to keyhole limpet hemocyanin (KLH) and mixed with QS-21 induce high-titer Ab (but no evidence of T-cell immunity) against MUC1 and MUC1-expressing tumor cells. Furthermore, these vaccinations conferred protection to these mice when they were challenged with MUC1-expressing tumor cells (90).

### Anti-CEA IgM

Carcinoembryonic Ag (CEA) was described in 1965 and was the first tumor Ag to be identified. CEA is a glycoprotein that belongs to the Ig family of genes and is detected in the serum of cancer patients by radioimmunoassay or ELISA. However, its clinical value is limited due to a high false-positive rate in normal populations and its low diagnostic sensitivity and specificity. Elevated CEA levels are not specific for BC (50% of cases), CEA is expressed in many types of neoplasia and is detected by anti-CEA IgM. Nevertheless, in breast tumors, CEA is more prevalent in ductal versus lobular carcinomas. CEA is found in patients with ductal carcinoma in situ, suggesting that it is an early marker of tumorigenesis (91,92).

### I antigen and IgM antibodies

The ABH blood group Ags, which are expressed in normal epithelial cells, are downregulated in various carcinomas. BC cells produce I Ag or I Ag-like substances that influence serum anti-I. I Ags are precursors to ABH and accumulate in cancer cells. The levels of I Ag rise in the serum of individuals with breast carcinoma, based on their anti-I scores and IgM concentrations. These alterations in cold hemagglutinins could be a host response to the production of I Ag by BC cells and is thus important in understanding immune modulation of breast carcinoma. However, the concentration of IgM in patients with BC was similar to that of controls (93).

### Anti-malignin antibodies in serum (AMAS)

Malignin is a 10-kDa polypeptide in the cytoplasmic and outer membranes of all malignant cells. Anti-malignin Ab (AMA) is an IgM that is spontaneously produced by the host against the oncoprotein malignin when neoplastic transformation occurs; because AMAs are IgM molecules, they are an indicator of 'early' transformation that is useful for the early detection of cancer.

Elevated AMA serum concentrations have been measured using a commercial reagent. The AMA test has a sensitivity and specificity of 95% on first determination and >99% on repeat measurements and is a promising diagnostic tool for the early detection of cancer, the monitoring of treatment responses, and the screening of asymptomatic populations (94).

In tumor marker assays with AMAs, CEA (CA15-3, CA19-9 and CA125) and biopsies were examined following a suspicious mammogram to determine whether tumor markers aided in the diagnosis and could be used to monitor residual disease. In the present study, by AMA test, 3 of 154 healthy volunteers were AMA-positive. After further examination, 2 were positive for cancer and 1 had a history of ulcerative colitis. Tumor biopsies of 43 suspicious patients by mammography revealed that 32 were cancerous and 11 were benign by pathology. Furthermore, 31 of 32 cancer patients were positive for AMA versus 4 of 11 pathological benign cases (95).

### Sialyl Tn antigen and IgM antibodies

Several structurally similar blood group-related carbohydrate Ags, including Thomsen-Freidenreich (TF), Tn, and sialyl Tn (sTn), that are attached to the protein backbones of glycoproteins are promising targets, based on their widespread distribution on the cell surface of human tumors. The disaccharide sTn [NeuAca (2→6) GalNAca-0-ser/Thr] is O-linked with serine and threonine residues on mucins. Greater sTn expression in tumors might be linked to a poorer prognosis in BC. Abnormal glycosylation of tumor cell MUCs results in shorter and fewer carbohydrate chains, increasing the exposure of Ags, such as sTn, which might upregulate sTn in tumors compared with normal cells.

The induction of IgM and IgG against synthetic sTn(c) was measured before and after immunization with clustered sTn-KLH [sTn(c)-KLH] conjugate plus QS-21 in 27 patients with BC, all of whom developed significant IgM and IgG titers against sTn(c). Furthermore, IgM reactivity against LSC tumor cells was observed in patients, indicating the production of IgM and IgG. Thus, immunization with sTn(c)-KLH conjugate plus QS-21 is well tolerated and immunogenic in high-risk patients with BC (96).

### IgM and IgG complexes

The occurrence of circulating immune complexes (CICs) is considered a marker of tumor burden. CICs are formed by Abs or Ags with and without complement. CICs that comprise two classes of Ig (Ig-Ig) or an Ig class of and C3 (Ig-C) are collectively referred to as two-component-determined CICs (TCICs). Ig/Ig TCICs may reveal alterations in immune regulation in patients. IgM and IgG TCICs were measured in the sera of patients with BC, in whom IgM/IgG TCICs and IgG/IgM TCICs were detected. Downregulation of IgM/IgG TCICs was a common feature in patients, whereas IgG/IgM TCIC levels were significantly higher, lower, or unchanged with respect to the control. Total serum IgM differed significantly in BC patients (1.20±0.94 mg/ml) vs. healthy controls (0.99±0.53 mg/ml). These results suggested that IgM and IgG TCICs have a significant function in immune regulation during the course of malignancies and are hallmarks of cancer pathogenesis. Decreased IgM/IgG TCIC levels, accompanied by IgG/IgM-TCICs, constitute a peculiar trait in malignancies (97).

## 6. Conclusions

Certain IgMs, natural and adaptive, have been isolated from the tumors of patients with cancer; they eliminate BC tumors through various mechanisms, such as apoptosis and complement. Natural IgM has a direct cytotoxic effect on tumor cells, it recognizes tumor-modified cell surfaces that develop during tumorigenesis, and it activates complement to destroy nascent transformed cells. The first Ig that is produced after an immune challenge is adaptive IgM, which must be considered as an early diagnostic tool. Auto-Abs that target TAAs could serve as molecular signatures for the early diagnosis and prognosis of patients with BC by serology to increase the sensitivity and specificity of diagnostic markers for BC patients. Most studies have focused on the development of IgG-like biomarkers for BC treatment; however, IgG is subject to immunoregulation, which can manifest as immunosuppression, whereas natural IgM is not. IgM as a diagnostic tool can be coupled with early mammography, magnetic resonance imaging, or Doppler ultrasound to detect cancer.
